# Repurposing mesalazine against cardiac fibrosis in vitro

**DOI:** 10.1007/s00210-020-01998-9

**Published:** 2020-10-16

**Authors:** Maximilian Hoffmann, Theresa A. Kant, Ramona Emig, Johanna S. E. Rausch, Manja Newe, Mario Schubert, Karolina Künzel, Luise Winter, Erik Klapproth, Rémi Peyronnet, Ursula Ravens, Ali El-Armouche, Stephan R. Künzel

**Affiliations:** 1 Institute of Pharmacology and Toxicology, Faculty of Medicine Carl Gustav Carus, Technische Universität Dresden, Fiedlerstraße 42, 01309 Dresden, Germany; 2 Institute for Experimental Cardiovascular Medicine, University Heart Center Freiburg-Bad Krozingen, Medical Center-University of Freiburg, Freiburg, Germany; 3 Faculty of Medicine, University of Freiburg, Freiburg, Germany; 4 CIBSS Centre for Integrative Biological Signalling Studies, University of Freiburg, Freiburg, Germany; 5 Faculty of Biology, University of Freiburg, Freiburg, Germany

**Keywords:** Myofibroblasts, Collagen, Phenoconversion, Cytoskeleton, Fibrosis mechanisms

## Abstract

Cardiovascular diseases are exacerbated and driven by cardiac fibrosis. TGFβ induces fibroblast activation and differentiation into myofibroblasts that secrete excessive extracellular matrix proteins leading to stiffening of the heart, concomitant cardiac dysfunction, and arrhythmias. However, effective pharmacotherapy for preventing or reversing cardiac fibrosis is presently unavailable. Therefore, drug repurposing could be a cost- and time-saving approach to discover antifibrotic interventions. The aim of this study was to investigate the antifibrotic potential of mesalazine in a cardiac fibroblast stress model. TGFβ was used to induce a profibrotic phenotype in a human cardiac fibroblast cell line. After induction, cells were treated with mesalazine or solvent control. Fibroblast proliferation, key fibrosis protein expression, extracellular collagen deposition, and mechanical properties were subsequently determined. In response to TGFβ treatment, fibroblasts underwent a profound phenoconversion towards myofibroblasts, determined by the expression of fibrillary αSMA. Mesalazine reduced differentiation nearly by half and diminished fibroblast proliferation by a third. Additionally, TGFβ led to increased cell stiffness and adhesion, which were reversed by mesalazine treatment. Collagen 1 expression and deposition—key drivers of fibrosis—were significantly increased upon TGFβ stimulation and reduced to control levels by mesalazine. SMAD2/3 and ERK1/2 phosphorylation, along with reduced nuclear NFκB translocation, were identified as potential modes of action. The current study provides experimental pre-clinical evidence for antifibrotic effects of mesalazine in an in vitro model of cardiac fibrosis. Furthermore, it sheds light on possible mechanisms of action and suggests further investigation in experimental and clinical settings.

**Electronic supplementary material:**

The online version of this article (10.1007/s00210-020-01998-9) contains supplementary material, which is available to authorized users.

## Introduction

Fibrosis is the excessive deposition of extracellular matrix (ECM) proteins, leading to organ dysfunction, morbidity and finally death. Worldwide, the burden of fibrosis is substantial, as 25% of the population are affected and approximately 45% of deaths in the Western world can be attributed to diseases involving fibroproliferation (Artlett 2012; Zhao et al. 2020).

The human heart is particularly vulnerable to fibrotic remodeling, as lost cardiomyocytes do not regenerate and thus are replaced by ECM proteins (Uygur and Lee 2016). Therefore, most cardiovascular diseases are accompanied by fibrosis (Murtha et al. 2017; Hinderer and Schenke-Layland 2019). In health, cardiac fibroblasts safeguard the ECM homeostasis by well-balanced secretion and degradation of ECM proteins, ensuring optimal tissue mechanical properties. Thereby, they protect the heart from rupture due to high mechanical load without negatively affecting cardiac function (Tallquist and Molkentin 2017). In disease, however, activating stimuli such as transforming growth factor beta 1 (TGFβ) induce a phenotypic transition of fibroblasts towards α-smooth muscle actin (αSMA)-positive myofibroblasts (Baum and Duffy 2011; Tallquist and Molkentin 2017), which excessively secrete collagen and release cytokines leading to local inflammation, cardiac dysfunction, and arrhythmias such as atrial fibrillation (Jalife and Kaur 2015; Künzel et al. 2020).

Recent clinical therapeutic approaches towards cardiac fibrosis concentrate on the modulation of the renin-angiotensin-aldosterone system with angiotensin-converting enzyme inhibitors, angiotensin-II receptor subtype-1 blockers, and aldosterone or renin inhibitors (Jia et al. 2018; Park et al. 2019). Furthermore, inflammation modulators targeting tumor necrosis factor alpha (e.g., infliximab), statins like rosuvastatin and peroxisomal proliferator–activated receptor agonists like fenofibrate have been tested to ameliorate cardiac fibrosis but failed to provide convincing results in several clinical studies (Fang et al. 2017). As of today, there are no drugs available that reliably prevent or substantially reverse fibrosis (Zhao et al. 2020).

Current research has focused on the experimental modulation of the TGFβ, extracellular signal-regulated kinases 1 and 2 (ERK1/2), and SMAD2/3 pathways in cardiac (myo)fibroblasts (Evans et al. 2003; Fan and Guan 2016; Khalil et al. 2017; Luo et al. 2017). Given the substantial costs and extensive timeline of de novo drug development to specifically modulate these pathways, repurposing of established compounds with known safety profiles could be an attractive and low-risk approach to providing antifibrotic therapy (Paul et al. 2010; Sertkaya et al. 2016; Pushpakom et al. 2019).

Aminosalicylates, like aspirin, have shown promising results in the experimental treatment of cardiac fibrosis (Liu et al. 2017). However, the translational value of this finding might be limited because dose-dependent systemic side effects such as bleeding or gastric ulcer are common with aspirin (Weil et al. 1995; Huang et al. 2011). Mesalazine (5-aminosalicylic acid) is structurally comparable to aspirin (Desreumaux and Ghosh 2006), but can be administered in high daily doses with good tolerability (Clemett and Markham 2000). While its mode of action is still under debate, experimental evidence suggests that orally administered mesalazine reduces the expression of profibrotic cytokines (Ramadan et al. 2018), predisposing the compound for further investigation into antifibrotic drug repurposing. In this study, we investigate the effect of mesalazine on fibrotic phenotype conversion of cardiac fibroblasts, using a previously described in vitro model (Künzel et al. 2020).

## Materials and methods

### Cell culture

All experiments were performed with the recently in-house developed human atrial fibroblast cell line HAF-SRK01 (HAF) (Künzel et al. 2020) (RRID:CVCL_ZG36). Cells were cultured under controlled conditions (37 °C, 90% humidity, 5% CO_2_) in non-coated cell culture dishes (Sigma-Aldrich; USA; Techno Plastic Products, Switzerland) containing as culture medium Dulbecco’s modified Eagle’s medium with high glucose (4500 mg/L; Sigma-Aldrich, USA), 10% fetal calf serum, and 1% penicillin-streptomycin.

### TGFβ stress model and mesalazine treatment protocol

**Fig. 1:**
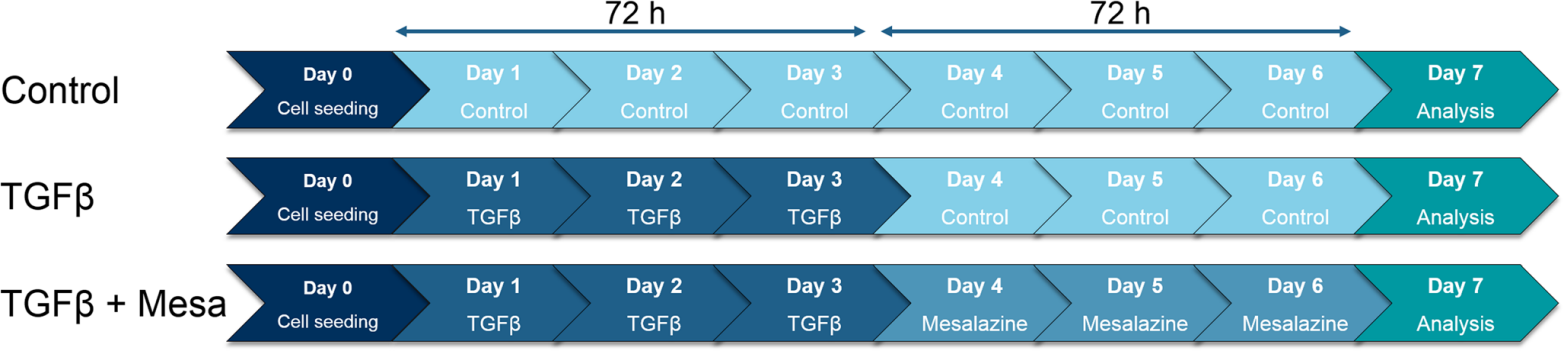
Schematic illustration of the TGFβ stress model and mesalazine treatment protocol

TGFβ is one of the most potent inductors of virtually all types of fibrosis (Rockey et al. 2015). Unless stated otherwise, 24 h after seeding, the cells were stimulated with TGFβ (10 ng/mL in medium) (100-21C, Peprotech, USA) for 72 h, followed by either 72 h of medium (solvent control) or 10 mmol/L mesalazine (A3537, Sigma-Aldrich, USA) solved in medium (Fig. 1). TGFβ treatment followed by solvent control is stated as “TGFβ” in the results, whereas TGFβ treatment followed by mesalazine is stated as “TGFβ + Mesa.” Medium and drugs (TGFβ and mesalazine) were changed daily.

### Functional fibroblast characterization

#### Proliferation

As described above, 1 × 10^4^ cells/well were seeded in 12-well plates with daily change of medium and drugs. Cells were harvested and counted after 5 and 10 days using 0.25% trypsin and a Buerker counting chamber. Results were calculated as cells × 10^4^/mL.

#### Myofibroblast differentiation

To evaluate myofibroblast differentiation, immunocytochemistry (ICC) for fibrillary αSMA was conducted as described previously (Poulet et al. 2016; Künzel et al. 2019). For this purpose, 0.5 × 10^4^ cells/well were seeded on glass coverslips in 24-well plates. Stimulation and treatment were performed as described above. Pictures of independent coverslips were randomly taken and the percentage of myofibroblasts was calculated in relation to the total number of counted nuclei. A minimum of 50 cells/coverslip was analyzed. Table 1 provides the primary and secondary antibodies that were used to detect the proteins of interest.

**Table 1:** Antibodies

Protein	Dilution	Conjugate/source	Product-Nr.	Usage
Primary antibodies				
αSMA	1:200 (ICC) 1:1000 (WB)	Mouse	A5228	ICC^1^ and WB^2^
Collagen 1	1:10.000	Rabbit	ab34710	WB
SMAD 2/3	1:1000	Rabbit	#3102	WB
Phospho-SMAD 2	1:1000	Rabbit	#8828	WB
ERK 1/2 (p42/44)	1:1000	Rabbit	#9102	WB
Phospho-ERK 1/2 (p42/44)	1:1000	Rabbit	#9101	WB
EEF2	1:50.000	Rabbit	ab40812	WB
GAPDH	1:50.000	Mouse	sc-365062	WB
Collagen 1 A1	1:100	Goat	MBS316282	ICC
NFκB (p65)	1:400	Rabbit	8242s	ICC
Secondary antibodies				
Goat-anti-mouse	1:10.000	Peroxidase	A3682	WB
Goat-anti-rabbit	1:10.000	Peroxidase	111-035-045	WB
Alexa fluor 546 (goat-anti-mouse)	1:400	Streptavidin	Z25004	ICC
Alexa fluor 546 (goat-anti-rabbit)	1:400	Streptavidin	Z25304	ICC
Alexa fluor 555 (donkey-anti-goat)	1:500	None	A32816	ICC

^1^Immunocytochemistry

^2^Western blot

#### NFκB translocation

Cells (0.5 × 10^4^/well) were seeded on coverslips in 24-well plates. The next day, the cells were cultured in drug-free medium as a control or stimulated with TGFβ (10 ng/mL) for 1 h, followed by drug-free medium or mesalazine (10 mmol/L) for an additional 2 h. After fixation, cells were permeabilized using Triton-X 100 (0.5%). Subsequently, ICC for NFκB was performed and nuclei were stained with DAPI. To evaluate the nuclear translocation of NFκB, images were quantified using CellProfiler™-Software (Broad Institute, Cambridge, USA) (McQuin et al. 2018). The results are presented as nuclear NFκB intensity normalized to nuclear area per analyzed cell.

### Collagen secretion and deposition

For assessment of collagen deposition on the growth surface, cells were first seeded on glass coverslips in standard culture medium at a density of 10 × 10^4^ cells/cm^2^. Twenty-four hours after seeding, the medium was changed to DMEM high glucose supplemented with 0.5% FCS, 1% penicillin/streptomycin, 0.5 mmol/L ascorbic acid (AAcid), and the indicated drugs. After 48 h, cells were fixed in 4% para-formaldehyde. Deposited collagen was visualized using ICC against collagen Iα1. Imaging was performed on a Leica SP8X line-scanning confocal microscope using a × 40 water-immersion objective. For image analysis, an in-house macro for FIJI (Schindelin et al. 2012) was used. In short, a maximal intensity projection of all planes was performed after background subtraction. A uniform intensity–based threshold was then used to identify the collagen-positive area. The overall collagen–covered area was then normalized to the number of nuclei in the respective image.

### Cell mechanical properties

The mechanical properties of cells in response to the indicated treatments were assessed using the Chiaro nanoindenter system (Optics11, Amsterdam, the Netherlands) as described previously (Emig et al. 2019). Briefly, a spherical tip with a 3-μm radius, attached to a calibrated cantilever with a spring constant of 0.03 N/m, was used to indent the sample while the bending of the cantilever was tracked by interferometry (Fig. 3a). On each cell, indentations were performed at three different places, excluding the nuclear region. The force applied to the cantilever was then calculated as the product of cantilever bending and spring constant. Sample indentations of 2–4 μm were performed at a displacement speed of 5 μm/s. The effective Young’s modulus (*E*_Eff_) was derived using a Hertzian model (red curve in Fig. 3b) for contact mechanics (Hertz 1882) under the assumption of a Poisson’s ratio of 0.5 for incompressible materials, commonly used for mechanical testing of cells and tissue (Guz et al. 2014). Throughout this manuscript, *E*_Eff_ is referred to as stiffness. Additionally, stress relaxation of the cells in response to indentation was calculated from the remaining load after holding the indenter at maximal compression for 2 s and is given as percentage of the maximal load. Cell adhesion to the indenter tip was estimated from the maximum negative force that was recorded upon cantilever retraction. Data analysis was performed using the DataViewer software (V2.3.0, Optics11, Amsterdam, the Netherlands) and in-house MatLab scripts (R2019a).

### SDS-PAGE, western blotting, and immunodetection

Protein was extracted from whole-cell lysates using radioimmunoprecipitation assay buffer (30 mM Tris, 0.5 mM EDTA, 150 mM NaCl, 1% NP-40, 0.1% SDS) supplemented with 10% protease and phosphatase inhibitors (Roche, Switzerland). To ascertain protein concentration, a bicinchoninic acid kit (Thermo Fischer, USA) was used. Western blots were performed as described previously (El-Armouche et al. 2008). A 20 μg of a whole-cell protein was separated on a 10% polyacrylamide gel and then transferred to a nitrocellulose membrane. Immunodetection was performed with a Fusion FX device (Vilber Lourmat Deutschland GmbH, Germany). Table 1 provides a list of antibodies and respective concentrations used in this study.

### Data analysis

For data analysis and graphic representation, Prism 8 (GraphPad, USA) was used. Data are presented as single data points and mean ± standard error of the mean (SEM). For comparisons between two conditions, Student’s *t* test was used with Welsh’s correction if appropriate. When comparing three or more conditions, a one-way ANOVA with Tukey posttest was performed. Two means were considered significantly different with *p* values < 0.05. A single asterisk (*), double asterisk (**), and triple asterisk (***) indicate *p* values below 0.05, 0.01, and 0.001, respectively.

## Results and discussion

### Mesalazine reverses fibrotic phenotype conversion

Resident cardiac fibroblasts are primarily responsible for the fibrotic remodeling of the heart (Khalil et al. 2017). As the availability of primary human cardiac fibroblasts is limited, we recently established the human atrial fibroblast cell line HAF-SRK01 (Künzel et al. 2020) (HAF), which was employed in this study to test potential antifibrotic effects of mesalazine. In our model, we induced a profibrotic phenotype by treating fibroblasts with 10 ng/mL TGFβ, a major regulator of fibrosis (Meng et al. 2016). Myofibroblast differentiation was determined by the expression of fibrillary αSMA which is characteristic for myofibroblasts (Baum and Duffy 2011).

**Fig. 2:**
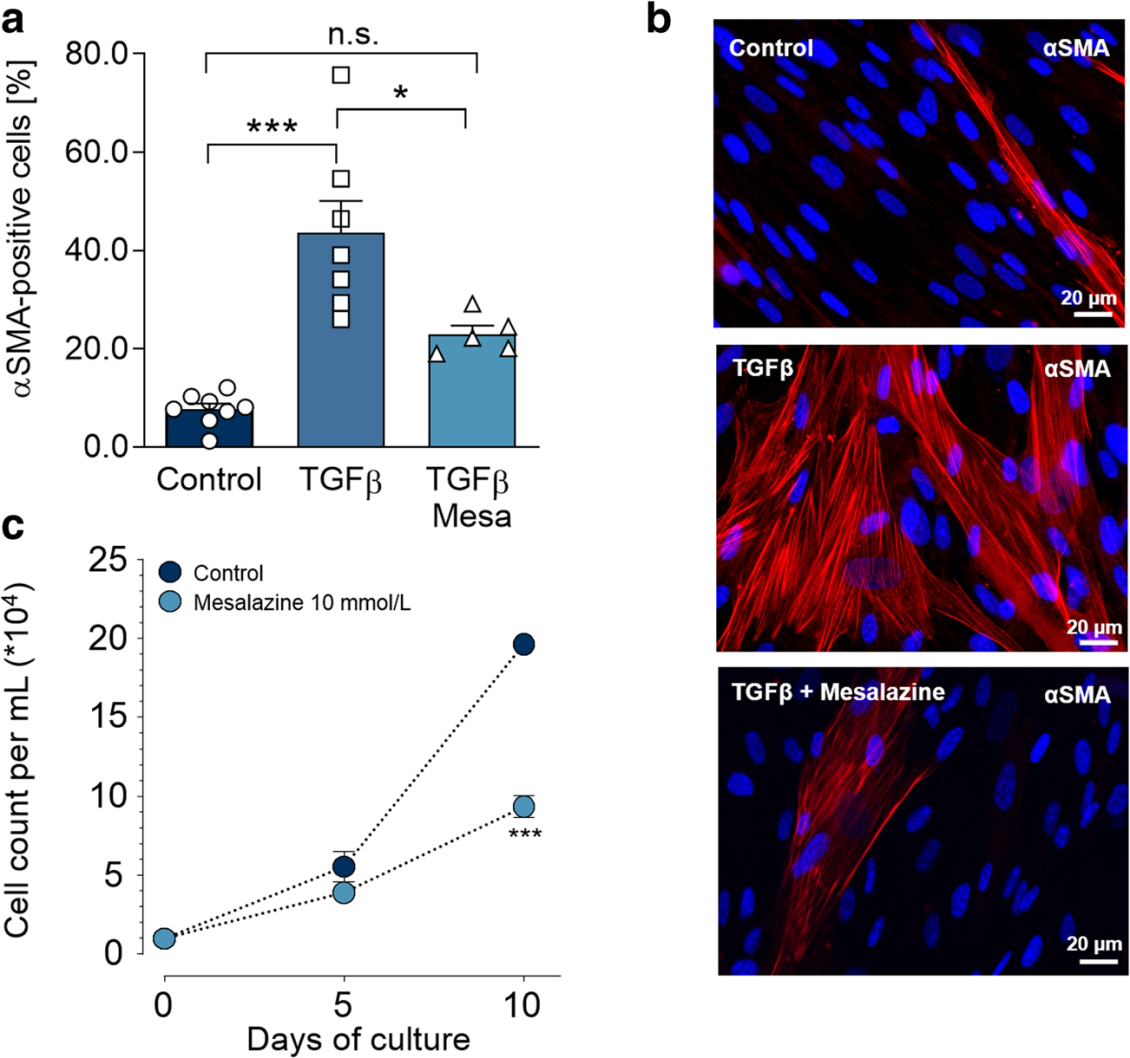
Mesalazine reduces TGFβ-induced myofibroblast differentiation and fibroblast proliferation. **a** Quantification of myofibroblast differentiation in HAF by immunohistochemical assessment of αSMA myofilaments (red). Myofibroblast differentiation was induced with 10 ng/mL TGFβ for 72 h. Subsequently cells were treated with mesalazine or solvent control (5 ≤ *n* ≤ 8-independent coverslips). **b** Representative immunofluorescence images for fibrillary αSMA (red), nuclei were stained with DAPI (blue), scale bars = 20 μm. **c** Fibroblast proliferation curves under control conditions and with 10 mmol/L mesalazine treatment which was continuously applied for the duration of the experiment (*n* = 6 per condition). Cells were harvested and counted after 5 and 10 days. The cell count was calculated in a resuspension volume of 1 mL

After the 72 h TGFβ induction phase, followed by 72 h in control medium, cultured fibroblasts displayed a fibrotic phenotype, as determined by ICC for fibrillary αSMA (Fig. 2a and b): 43.6 ± 6.5% of cells were identified as myofibroblasts, compared to 7.7 ± 1.2% in the absence of TGFβ treatment (*p* < 0.001). This fibrotic effect of TGFβ was reduced to levels that were not significantly different from control by mesalazine treatment, to 23.0 ± 1.8% (*p* < 0.05) (Fig. 2a and b).

Fibroblast proliferation is another prominent indicator of a profibrotic phenotype. In parallel to lowering αSMA expression, mesalazine treatment also significantly reduced proliferation to roughly half of that seen under control conditions (*P* < 0.001; Fig. 2c).

To control cardiac fibrosis, reduction of both excess fibroblast proliferation and myofibroblast differentiation are essential (Fan and Guan 2016). Our results are in line with previous studies showing general antiproliferative effects of mesalazine in mucosal cells of the large bowel and colorectal cancer cells (Reinacher-Schick et al. 2000; Carmine Stolfi and Francesco Pallone 2008). Effects on cardiac fibroblast proliferation and differentiation were previously unknown.

### Mesalazine affects mechanical properties of HAF

**Fig. 3:**
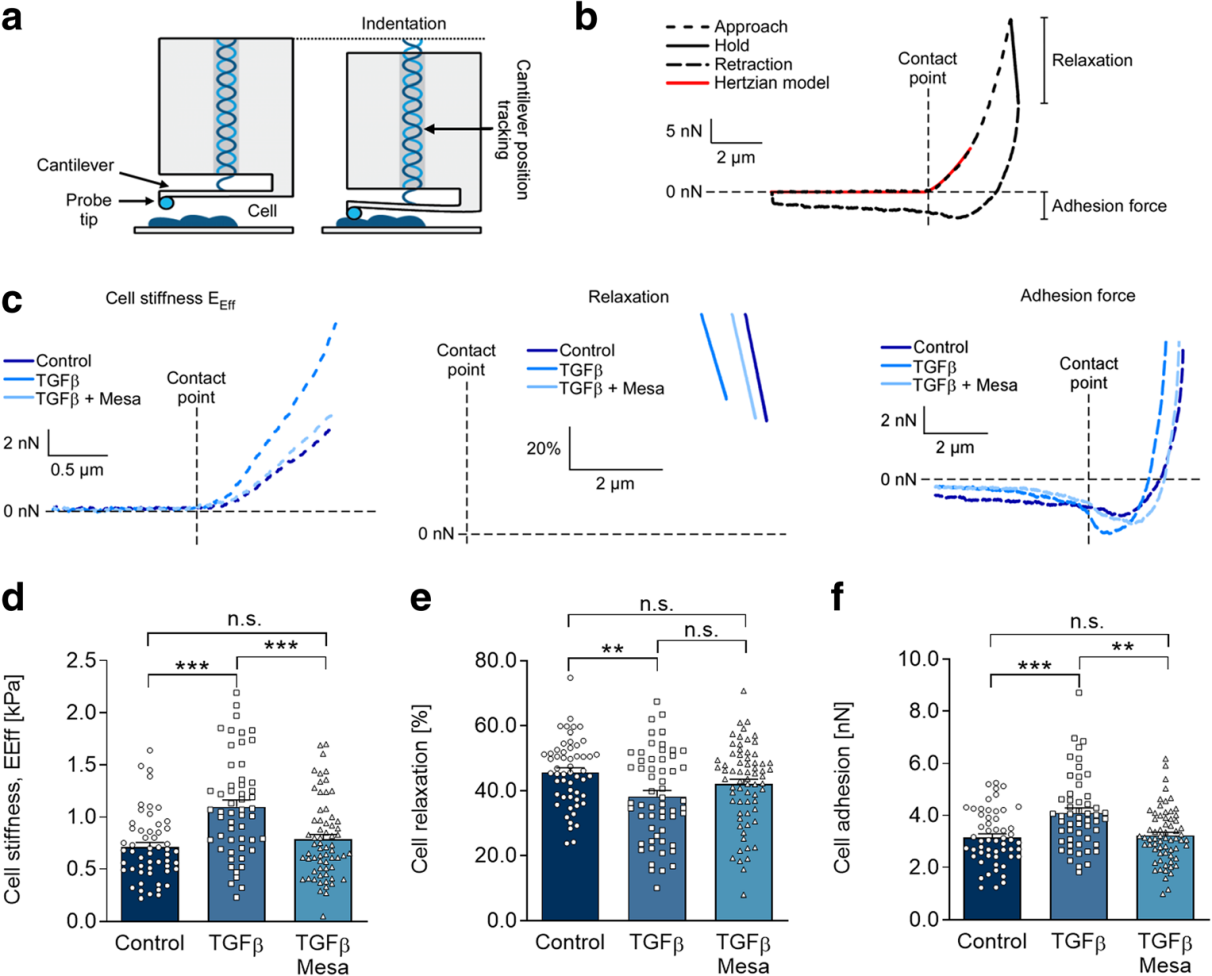
Mesalazine reverses TGFβ-induced changes in HAF mechanical properties. **a** Schematic representation of the nanoindenter probe before (left) and during (right) cell indentation, adapted from Optics11. **b** and **c** Representative load/indentation curve used to calculate cell stiffness (*E*_eff_), stress relaxation, and cell adhesion of individual HAF cultured under control conditions, with TGFβ (10 ng/mL) and with TGFβ followed by mesalazine (10 mmol/L) treatment (whole curve (b) and with each phase shown separately for the 3 conditions (c; note that relaxation is plotted as relative values). **d** Cell stiffness measured under control conditions, with TGFβ (10 ng/mL) and with TGFβ followed by mesalazine (10 mmol/L) treatment (*n* = 54, 54, and 62 respectively from 4 independent experiments). **e** Cell relaxation measured under control conditions, with TGFβ (10 ng/mL) and with TGFβ followed by mesalazine (10 mmol/L) treatment (*n* = 55, 57, and 67 respectively from 4 independent experiments). **f** Cell adhesion measured under control conditions, with TGFβ (10 ng/mL) and with TGFβ followed by mesalazine (10 mmol/L) treatment (*n* = 55, 54, and 67 respectively from 4 independent experiments)

After finding that mesalazine treatment affects the expression of αSMA in HAF, we aimed to assess its effect on the remodeling of the cytoskeleton from a more general perspective. To do so, we analyzed the mesalazine effects on cell mechanical properties, which depend directly on cytoskeleton composition and organization. When fibroblasts are activated, the cytoskeleton is remodeled, which contributes to a number of cell functions (Hinz et al. 2001; Hinz et al. 2019). Using nanoindentation (Fig. 3a), load indentation curves (Fig. 3b and c) were obtained and used to quantify cell stiffness, relaxation and adhesion (Fig. 3c–f) Compared to control conditions, average cell stiffness increased from 0.71 to 1.10 kPa in response to TGFβ (*p* < 0.001). Subsequent mesalazine treatment reduced average cell stiffness to 0.83 kPa (*p* < 0.001), a level not different from control conditions (Fig. 3 c left panel and d). In line with increased cell stiffness, stress relaxation of TGFβ-treated cells was lower than in control cells (38.3 vs. 45.7%, respectively; *p* < 0.01). In response to mesalazine treatment, stress relaxation was not significantly different from both the control condition and TGFβ, highlighting an increased scattering with more cells showing relaxation values not different from control (Fig. 3 c middle panel and e).

Fibrosis and myofibroblast differentiation have been linked to an increased presence of cell adhesion molecules (Yoshizaki et al. 2010; Schroer and Merryman 2015). Therefore, we assessed the adhesive properties of HAF in response to TGFβ stimulation and subsequent mesalazine treatment by determining the maximal negative force that was recorded during cantilever retraction. TGFβ treatment significantly increased the adhesion to the indenter tip compared to control (3.15 nN vs. 4.19 nN, *p* < 0.001; Fig. 3d). Mesalazine treatment reduced the adhesive force of the cells to 3.37 nN (*p* < 0.01), a level not significantly different from control conditions (Fig. 3 c right panel and d).

Our findings are in keeping with previous studies relating TGFβ signaling to cell adhesion via integrin signaling (Walsh and Young 2011, p. 1).

### Mesalazine ameliorates key fibrosis protein expression and collagen deposition

Collagen 1 is the predominant type of collagen in cardiac fibrosis (Zhao et al. 2020), and it was therefore used as a readout for determining the effectiveness of the antifibrotic intervention. Having demonstrated that mesalazine reverses TGFβ-induced changes in differentiation, proliferation, and cell mechanical properties, we studied the effects of mesalazine on intracellular collagen 1 and αSMA expression to extend the ICC observations.

**Fig. 4:**
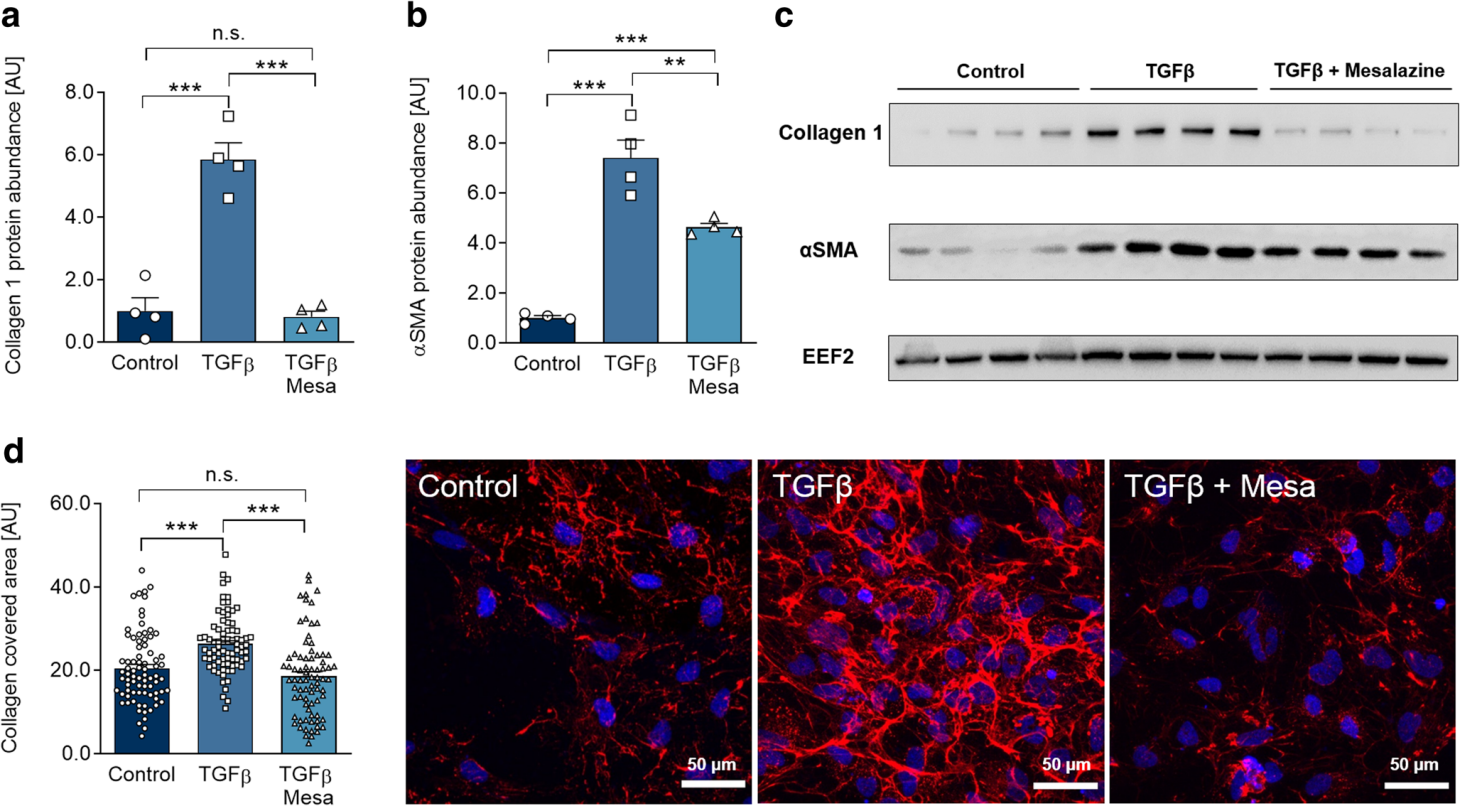
Mesalazine ameliorates key fibrosis protein expression and collagen deposition after TGFβ treatment. **a** Quantification of collagen 1 protein expression in HAF under control conditions, TGFβ and sequential TGFβ-mesalazine treatment (*n* = 4 per condition). **b** Quantification of αSMA protein expression in HAF fibroblasts under control conditions, TGFβ and sequential TGFβ-mesalazine treatment (*n* = 4 per condition). **c** Representative western blots for (a) and (b). EEF2, eukaryotic elongation factor 2. **d** Quantification and representative immunofluorescence images (collagen, red; DAPI-stained nuclei, blue) of extracellular collagen deposition of HAF under control conditions, TGFβ and sequential TGFβ-mesalazine treatment (77 ≤ *n* ≤ 70)

TGFβ treatment led to enhanced intracellular collagen 1 and αSMA protein expression by approximately 6- and 8-fold, respectively (*p* < 0.001). Mesalazine treatment abolished the TGFβ-induced increase in HAF intracellular collagen expression (*p* < 0.001; Fig. 4a–c) and reduced the level of αSMA protein expression (*p* < 0.01, Fig. 4a–c).

Extracellular matrix production is a complex process and tightly regulated at different levels. After finding that mesalazine treatment reduced intracellular collagen 1 expression, we determined if the deposition of fibrillary collagen 1 in the extracellular space was affected by mesalazine. ICC further revealed that TGFβ treatment of HAF increased extracellular collagen deposition (*p* < 0.001, Fig. 4d), which was also abolished by subsequent mesalazine treatment (*p* < 0.001, Fig. 4d).

### Mesalazine acts as a dual inhibitor of SMAD2/3 and ERK1/2 phosphorylation and reduces nuclear translocation of NFκB

Although mesalazine has been in clinical use for decades, its mode of action is still a matter of debate. Therefore, we aimed to identify mechanisms by which mesalazine exerts its antifibrotic effects in the experimental setting of in vitro fibrosis.

**Fig. 5:**
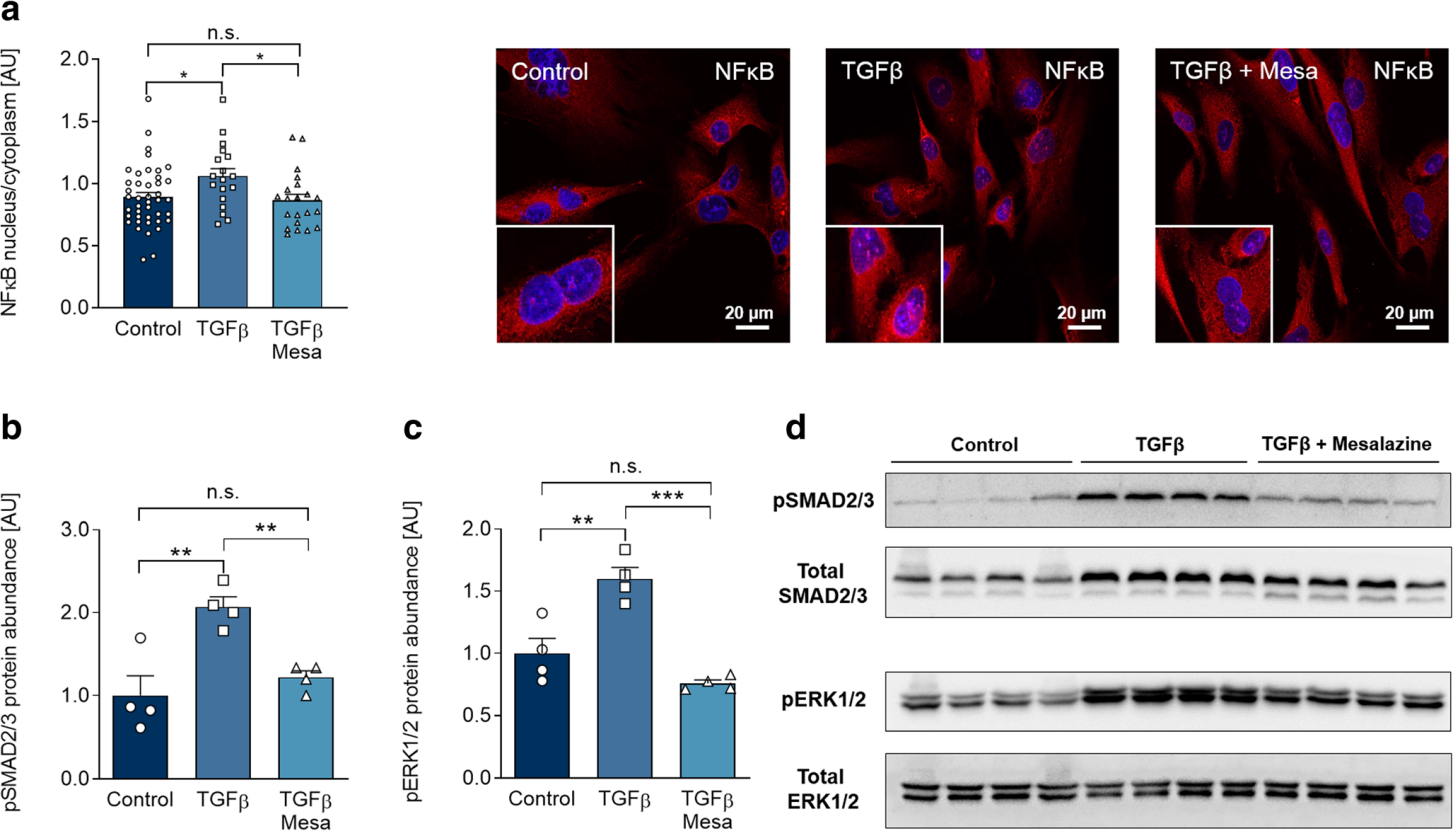
Mesalazine inhibits TGFβ-induced SMAD2/3 and ERK1/2 phosphorylation and reduces nuclear NFκB translocation. **a** Quantification and representative immunofluorescence images of NFκB (NFκB (p65) red; DAPI-stained nuclei blue) under control conditions, TGFβ and sequential TGFβ-mesalazine treatment (18 ≤ *n* ≤ 43 cells from each 4 independent coverslips). **b** Quantification of SMAD2/3 phosphorylation (pSMAD2/3) in HAF under control conditions, TGFβ and sequential TGFβ-mesalazine treatment (*n* = 4 per condition). **c** Quantification of ERK1/2 phosphorylation (p ERK1/2) in HAF fibroblasts under control conditions, TGFβ and sequential TGFβ-mesalazine treatment (*n* = 4 per condition). **d** Representative western blots for (b) and (c)

Inhibition of the proinflammatory NFκB-signaling pathway by mesalazine in inflammatory bowel disease is a widely accepted concept (Desreumaux and Ghosh 2006). We investigated whether NFκB translocation to the nucleus was affected by TGFβ and subsequent mesalazine treatment in cardiac fibroblasts. In response to TGFβ stimulation, we found a significant increase in nuclear NFκB, which was restored to control levels by mesalazine (*p* < 0.05, Fig. 5a). This is particularly interesting, since NFκB has been shown to induce myofibroblast differentiation and fibrosis in pulmonary and hepatic fibrosis (Luedde and Schwabe 2011; Dong and Ma 2019).

SMAD2/3 and ERK1/2 activation by TGFβ are central mechanisms of the fibrotic signaling cascade (Khalil et al. 2017; Khalil et al. 2017; Luo et al. 2017) and modulation of these pathways has been proposed to affect cardiac and non-cardiac fibrosis (Rosenbloom et al. 2013; Cheng et al. 2016; Khalil et al. 2017; Li et al. 2018). In a murine model of pressure overload, selective block of SMAD2/3 signaling drastically attenuated fibrosis (Khalil et al. 2017). Also, ERK1/2 inhibition attenuated fibrotic remodeling after myocardial infarction (Luo et al. 2017). Although the structurally similar aspirin has been shown to suppress ERK1/2 and SMAD2/3 signaling (Li et al. 2018; Zhang et al. 2020), systemic side effects might overshadow the potential benefits. For this reason, we explored the effects of mesalazine on SMAD2/3 and ERK1/2 activation.

TGFβ stimulation led to a significant increase of both SMAD2/3 and ERK1/2 phosphorylation (*p* < 0.01, Fig. 5b–d). After mesalazine treatment, SMAD2/3 and ERK1/2 phosphorylation were reduced to control levels (*p* < 0.01, Fig. 5b–d). These results underline the potential of repurposing mesalazine against cardiac fibrosis, as it restores SMAD2/3, ERK1/2, and NFκB homeostasis in cardiac fibroblasts, opening up new therapeutic directions.

### Suitability of other aminosalicylates against cardiac fibrosis?

Besides mesalazine, sulfasalazine is frequently used to treat inflammatory bowel disease and rheumatoid arthritis. Sulfasalazine consists of sulfapyridine and mesalazine, which both have been shown to exert anti-inflammatory effect (Chávez et al. 2012). Sulfasalazine prevented induced hepatic fibrosis in a rat model by inhibiting nuclear NFκB translocation and the TGFβ pathway (Chávez et al. 2012). Therefore, it could be hypothesized that antifibrotic effects are also conceivable in the heart. However, there is currently no data available to support or refute this hypothesis. Irrespective of these similarities to mesalazine, the contained sulfapyridine frequently causes allergic reactions and is considered responsible for most of the side effects of sulfasalazine (Thornton and Mason 2012). Moreover, severe hematological side effects (blood dyscrasias), hepatitis, and male infertility have been reported more frequently than with mesalazine alone (Toovey et al. 1981; Ransford and Langman 2002). In summary, mesalazine appears therefore to be the more promising drug and our results indicate that mesalazine alone is sufficient for significant antifibrotic effects in vitro.

### Study limitations

Using a cell line, beside its advantage of being phenotypically stable over many passages, always raises the question of how closely the immortalized cells are to native primary cardiac fibroblasts. Additionally, it is difficult to project towards systemic effects of drugs, judging from in vitro experiments. Table 2 summarizes common adverse effects of mesalazine used to treat inflammatory bowel disease (Ransford and Langman 2002; Klotz 2012; Böhm and Kruis 2014). Future research has to focus on the effects of mesalazine in vivo to determine cardiac antifibrotic benefits and potential systemic side effects. Despite these limitations, we are optimistic that mesalazine is a promising candidate for antifibrotic drug repurposing.

**Table 2:** Selected adverse effects of mesalazine

Event	Frequency
Nausea/vomiting	Common
Headache	Common
Abdominal pain	Common
Skin exanthema	Common
Interstitial nephritis	Less common
Pancreatitis	Less common
Blood dyscrasias	Rare

## Conclusion

**Fig. 6:**
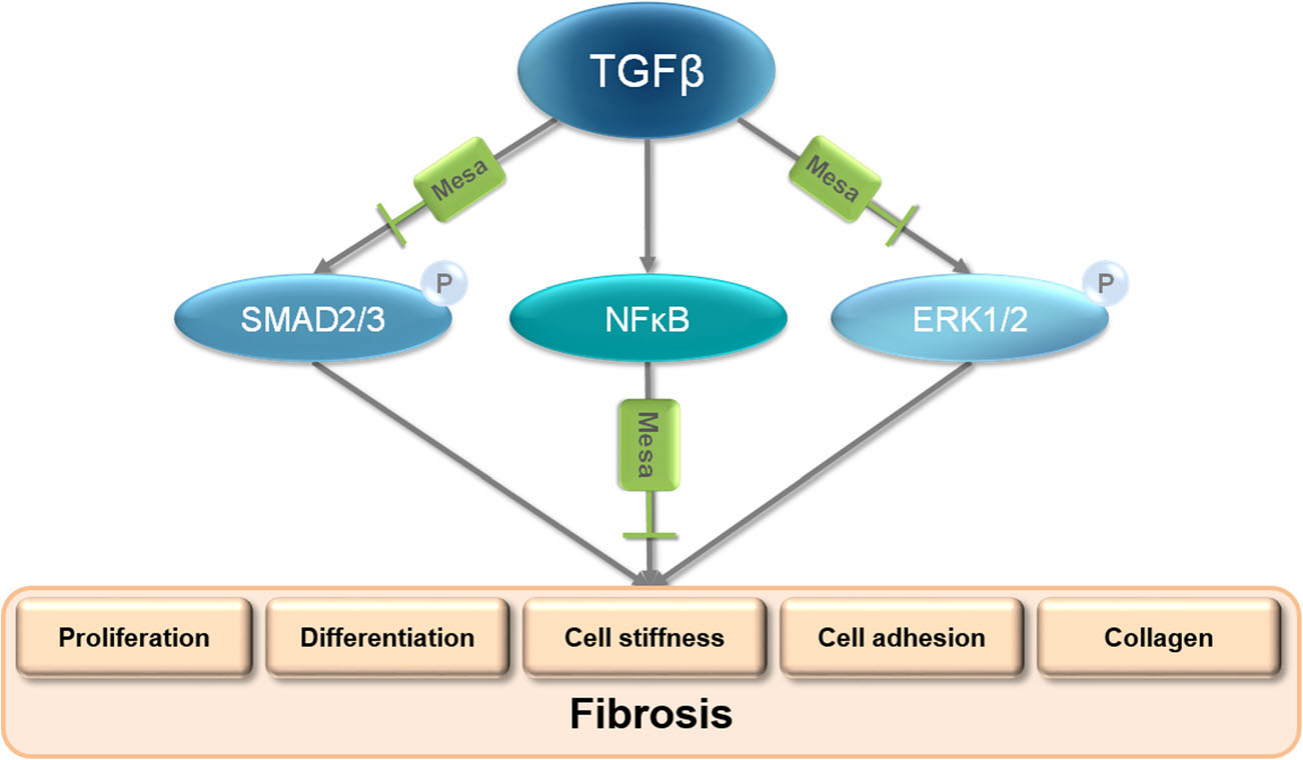
Schematic illustration of mesalazine’s proposed mechanism of antifibrotic action in fibroblasts. Upon stimulation with TGFβ, SMAD2/3, and ERK1/2 are activated, which is reflected by increased protein phosphorylation. Additionally, NFκB translocates from the cytosol to the nucleus, where it may induce fibrotic gene expression. Together, these mechanisms lead to myofibroblast differentiation and collagen deposition, which are hallmarks of fibrosis. Mesalazine treatment inhibits SMAD2/3 and ERK1/2 phosphorylation and prevents nuclear translocation of NFκB, making it an attractive candidate for antifibrotic intervention.

Here, we suggest that mesalazine may positively affect cardiac fibrosis—one of the most common causes of morbidity and mortality worldwide (Artlett 2012; Zhao et al. 2020). We found that mesalazine reduces fibroblast proliferation, myofibroblast differentiation, and collagen deposition after TGFβ induction. Furthermore, mesalazine ameliorated TGFβ-induced changes in fibroblast mechanical properties, such as cell stiffness, stress relaxation, and cell adhesion. Finally, we shed new light on the molecular mechanisms of mesalazine: we propose dual inhibition of SMAD2/3 and ERK1/2 phosphorylation as a novel concept by which mesalazine may prevent cardiac fibrosis (Fig. 6). Additionally, we were able to validate the accepted mechanism of inhibiting NFκB activity. With years of documented clinical use, a favorable risk profile and low cost, we believe that mesalazine is an exciting candidate for further studies on drug repurposing to finally treat fibrosis.

## Electronic supplementary material

ESM 1 (PPTX 317 kb)

ESM 2 (PZF 1883 kb)

## Data Availability

All data analyzed during this study are included in this article. All immunoblots are presented completely.

## References

[CR1] Artlett CM (2012) The role of the NLRP3 inflammasome in fibrosis. Open Rheumatol J 6(1). 10.2174/1874312901206010080 10.2174/1874312901206010080 PMC3395884 22802905

[CR2] Baum J, Duffy HS (2011). Fibroblasts and myofibroblasts: what are we talking about?. J Cardiovasc Pharmacol.

[CR3] Böhm SK, Kruis W (2014). Long-term efficacy and safety of once-daily mesalazine granules for the treatment of active ulcerative colitis. Clin Exp Gastroenterol.

[CR4] Carmine Stolfi RP, Francesco Pallone GM (2008). Molecular basis of the potential of mesalazine to prevent colorectal cancer. World J Gastroenterol.

[CR5] Chávez E, Castro-Sánchez L, Shibayama M, Tsutsumi V, Moreno M, Muriel P (2012). Sulfasalazine prevents the increase in TGF-β, COX-2, nuclear NFκB translocation and fibrosis in CCl4-induced liver cirrhosis in the rat. Hum Exp Toxicol.

[CR6] Cheng M, Wu G, Song Y, Wang L, Tu L, Zhang L, Zhang C (2016). Celastrol-induced suppression of the MiR-21/ERK signalling pathway attenuates cardiac fibrosis and dysfunction. CPB.

[CR7] Clemett D, Markham A (2000). Prolonged-release mesalazine: a review of its therapeutic potential in ulcerative colitis and Crohn’s disease. Drugs.

[CR8] Desreumaux P, Ghosh S (2006). Review article: mode of action and delivery of 5-aminosalicylic acid – new evidence. Aliment Pharmacol Ther.

[CR9] Dong J, Ma Q (2019). In vivo activation and pro-fibrotic function of NF-κB in fibroblastic cells during pulmonary inflammation and fibrosis induced by carbon nanotubes. Front Pharmacol.

[CR10] El-Armouche A, Wittköpper K, Degenhardt F, Weinberger F, Didié M, Melnychenko I, Grimm M, Peeck M, Zimmermann WH, Unsöld B, Hasenfuss G, Dobrev D, Eschenhagen T (2008). Phosphatase inhibitor-1-deficient mice are protected from catecholamine-induced arrhythmias and myocardial hypertrophy. Cardiovasc Res.

[CR11] Emig R, Zgierski-Johnston CM, Beyersdorf F, Rylski B, Ravens U, Weber W, Kohl P, Hörner M, Peyronnet R (2019). Human atrial fibroblast adaptation to heterogeneities in substrate stiffness. Front Physiol.

[CR12] Evans RA, Tian YC, Steadman R, Phillips AO (2003). TGF-beta1-mediated fibroblast-myofibroblast terminal differentiation-the role of Smad proteins. Exp Cell Res.

[CR13] Fan Z, Guan J (2016). Antifibrotic therapies to control cardiac fibrosis. Biomater Res.

[CR14] Fang L, Murphy AJ, Dart AM (2017) A clinical perspective of anti-fibrotic therapies for cardiovascular disease. Front Pharmacol 8. 10.3389/fphar.2017.00186 10.3389/fphar.2017.00186 PMC5382201 28428753

[CR15] Guz N, Dokukin M, Kalaparthi V, Sokolov I (2014). If cell mechanics can be described by elastic modulus: study of different models and probes used in indentation experiments. Biophys J.

[CR16] Hertz H (1882). Ueber die Berührung fester elastischer Körper. Journal für die reine und angewandte Mathematik.

[CR17] Hinderer S, Schenke-Layland K (2019). Cardiac fibrosis – a short review of causes and therapeutic strategies. Adv Drug Deliv Rev.

[CR18] Hinz B, Celetta G, Tomasek JJ, Gabbiani G, Chaponnier C (2001). Alpha-smooth muscle actin expression upregulates fibroblast contractile activity. Mol Biol Cell.

[CR19] Hinz B, McCulloch CA, Coelho NM (2019). Mechanical regulation of myofibroblast phenoconversion and collagen contraction. Exp Cell Res.

[CR20] Huang ES, Strate LL, Ho WW, Lee SS, Chan AT (2011). Long-term use of aspirin and the risk of gastrointestinal bleeding. Am J Med.

[CR21] Jalife J, Kaur K (2015). Atrial remodeling, fibrosis, and atrial fibrillation. Trend Cardiovasc Med.

[CR22] Jia G, Aroor AR, Hill MA, Sowers JR (2018). Role of RAAS activation in promoting cardiovascular fibrosis and stiffness. Hypertension.

[CR23] Khalil H, Kanisicak O, Prasad V, Correll RN, Fu X, Schips T, Vagnozzi RJ, Liu R, Huynh T, Lee S-J, Karch J, Molkentin JD (2017). Fibroblast-specific TGF-β–Smad2/3 signaling underlies cardiac fibrosis. J Clin Invest.

[CR24] Klotz U (2012). The pharmacological profile and clinical use of mesalazine (5-aminosalicylic acid). Arzneimittelforschung.

[CR25] Künzel SR, Rausch JSE, Schäffer C, Hoffmann M, Künzel K, Klapproth E, Kant T, Herzog N, Küpper J, Lorenz K, Dudek S, Emig R, Ravens U, Rog-Zielinska EA, Peyronnet R, El-Armouche A (2020). Modeling atrial fibrosis in vitro—generation and characterization of a novel human atrial fibroblast cell line. FEBS Open Bio.

[CR26] Künzel SR, Schaeffer C, Sekeres K, Mehnert CS, Schacht Wall SM, Newe M, Kämmerer S, El-Armouche A (2019) Ultrasonic-augmented Primary adult fibroblast isolation. J Vis Exp 149. 10.3791/59858 10.3791/59858 31403625

[CR27] Li X, Wang G, QiLi M, Liang H, Li T, Feng Y, Zhang Y, Liu X, Qian M, Xu B, Shen Z, Gitau SC, Zhao D, Shan H, E X (2018). Aspirin reduces cardiac interstitial fibrosis by inhibiting Erk1/2-serpine2 and P-Akt signalling pathways. CPB.

[CR28] Liu P-P, Liu H-H, Sun S-H, Shi X-X, Yang W-C, Su G-H, Zhao J (2017). Aspirin alleviates cardiac fibrosis in mice by inhibiting autophagy. Acta Pharmacol Sin.

[CR29] Luedde T, Schwabe RF (2011). NF-κB in the liver--linking injury, fibrosis and hepatocellular carcinoma. Nat Rev Gastroenterol Hepatol.

[CR30] Luo S, Hieu TB, Ma F, Yu Y, Cao Z, Wang M, Wu W, Mao Y, Rose P, Law BY-K, Zhu YZ (2017). ZYZ-168 alleviates cardiac fibrosis after myocardial infarction through inhibition of ERK1/2-dependent ROCK1 activation. Sci Rep.

[CR31] McQuin C, Goodman A, Chernyshev V, Kamentsky L, Cimini BA, Karhohs KW, Doan M, Ding L, Rafelski SM, Thirstrup D, Wiegraebe W, Singh S, Becker T, Caicedo JC, Carpenter AE (2018). CellProfiler 3.0: next-generation image processing for biology. PLoS Biol.

[CR32] Meng X, Nikolic-Paterson DJ, Lan HY (2016). TGF-β: the master regulator of fibrosis. Nat Rev Nephrol.

[CR33] Murtha LA, Schuliga MJ, Mabotuwana NS, Hardy SA, Waters DW, Burgess JK, Knight DA, Boyle AJ (2017) The processes and mechanisms of cardiac and pulmonary fibrosis. Front Physiol 8. 10.3389/fphys.2017.00777 10.3389/fphys.2017.00777 PMC5643461 29075197

[CR34] Park S, Nguyen NB, Pezhouman A, Ardehali R (2019). Cardiac fibrosis: potential therapeutic targets. Transl Res.

[CR35] Paul SM, Mytelka DS, Dunwiddie CT, Persinger CC, Munos BH, Lindborg SR, Schacht AL (2010). How to improve R&D productivity: the pharmaceutical industry’s grand challenge. Nat Rev Drug Discov.

[CR36] Poulet C, Künzel, Stephan, Büttner E, Lindner D, Westermann D, Ravens U (2016) Altered physiological functions and ion currents in atrial fibroblasts from patients with chronic atrial fibrillation. Phys Rep 4(2). 10.14814/phy2.12681 10.14814/phy2.12681 PMC4760386 26811054

[CR37] Pushpakom S, Iorio F, Eyers PA, Escott KJ, Hopper S, Wells A, Doig A, Guilliams T, Latimer J, McNamee C, Norris A, Sanseau P, Cavalla D, Pirmohamed M (2019). Drug repurposing: progress, challenges and recommendations. Nat Rev Drug Discov.

[CR38] Ramadan A, Afifi N, Yassin NZ, Abdel-Rahman RF, Abd El-Rahman SS, Fayed HM (2018). Mesalazine, an osteopontin inhibitor: the potential prophylactic and remedial roles in induced liver fibrosis in rats. Chem Biol Interact.

[CR39] Ransford RAJ, Langman MJS (2002). Sulphasalazine and mesalazine: serious adverse reactions re-evaluated on the basis of suspected adverse reaction reports to the Committee on Safety of Medicines. Gut.

[CR40] Reinacher-Schick A, Seidensticker F, Petrasch S, Reiser M, Philippou S, Theegarten D, Freitag G, Schmiegel W (2000). Mesalazine changes apoptosis and proliferation in normal mucosa of patients with sporadic polyps of the large bowel. Endoscopy.

[CR41] Rockey DC, Bell PD, Hill JA. 2015. Fibrosis — a common pathway to organ injury and failure.: 10.1056/NEJMra1300575.

[CR42] Rosenbloom J, Mendoza FA, Jimenez SA (2013). Strategies for anti-fibrotic therapies. Biochim Biophys Acta (BBA) - Mol Basis Dis.

[CR43] Schindelin J, Arganda-Carreras I, Frise E, Kaynig V, Longair M, Pietzsch T, Preibisch S, Rueden C, Saalfeld S, Schmid B, Tinevez J-Y, White DJ, Hartenstein V, Eliceiri K, Tomancak P, Cardona A (2012). Fiji: an open-source platform for biological-image analysis. Nat Methods.

[CR44] Schroer AK, Merryman WD (2015). Mechanobiology of myofibroblast adhesion in fibrotic cardiac disease. J Cell Sci.

[CR45] Sertkaya A, Wong H-H, Jessup A, Beleche T (2016). Key cost drivers of pharmaceutical clinical trials in the United States. Key cost drivers of pharmaceutical clinical trials in the United.

[CR46] Tallquist MD, Molkentin JD (2017). Redefining the identity of cardiac fibroblasts. Nat Rev Cardiol.

[CR47] Thornton C, Mason JC (2012) Chapter 16 - Drugs for inflammation and joint disease. In: Bennett PN, Brown MJ, Sharma P (eds) Clinical Pharmacology, Eleventh edn. Churchill Livingstone, Oxford, pp 240–259. 10.1016/B978-0-7020-4084-9.00055-0

[CR48] Toovey S, Hudson E, Hendry WF, Levi AJ (1981). Sulphasalazine and male infertility: reversibility and possible mechanism. Gut.

[CR49] Uygur A, Lee RT (2016). Mechanisms of cardiac regeneration. Dev Cell.

[CR50] Walsh JE, Young MRI (2011). TGF-beta regulation of focal adhesion proteins and motility of premalignant oral lesions via protein phosphatase 1. Anticancer Res.

[CR51] Weil J, Colin-Jones D, Langman M, Lawson D, Logan R, Murphy M, Rawlins M, Vessey M, Wainwright P (1995). Prophylactic aspirin and risk of peptic ulcer bleeding. BMJ.

[CR52] Yoshizaki A, Yanaba K, Iwata Y, Komura K, Ogawa A, Akiyama Y, Muroi E, Hara T, Ogawa F, Takenaka M, Shimizu K, Hasegawa M, Fujimoto M, Tedder TF, Sato S (2010). Cell adhesion molecules regulate fibrotic process via Th1/Th2/Th17 cell balance in a bleomycin-induced scleroderma model. J Immunol.

[CR53] Zhang Z, Li S, Deng J, Yang S, Xiang Z, Guo H, Xi H, Sang M, Zhang W (2020). Aspirin inhibits endometrial fibrosis by suppressing the TGF-β1-Smad2/Smad3 pathway in intrauterine adhesions. Int J Mol Med.

[CR54] Zhao X, Kwan JYY, Yip K, Liu PP, Liu F-F (2020). Targeting metabolic dysregulation for fibrosis therapy. Nat Rev Drug Discov.

