# Supplementary figures and images for: Repurposing mesalazine against cardiac fibrosis in vitro
Source: Naunyn Schmiedebergs Arch Pharmacol. 2020 Oct 16;394(3):533–43. doi: 10.1007/s00210-020-01998-9 (PMC7892689; doi:10.1007/s00210-020-01998-9)

## Slide 1
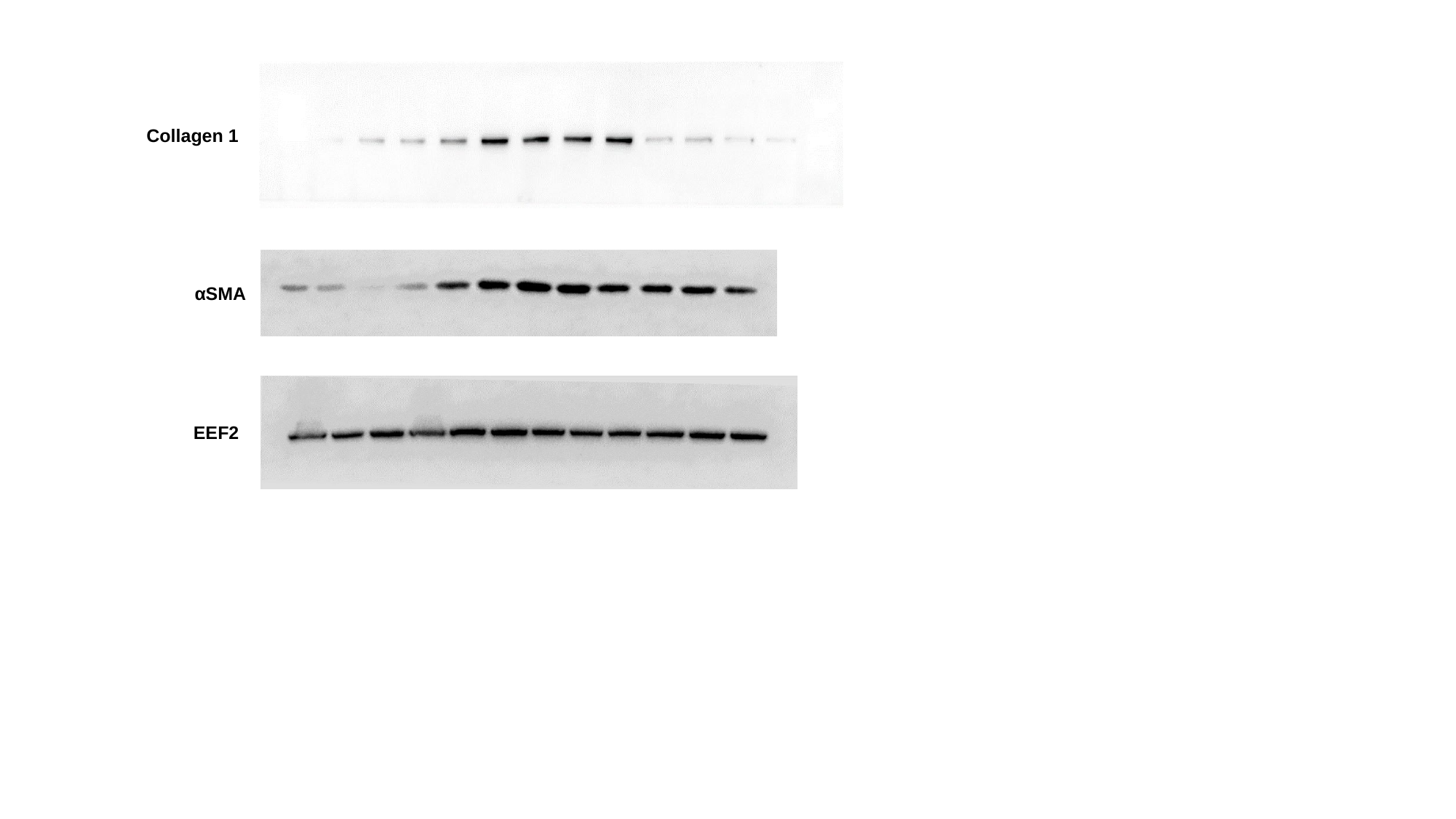

Collagen 1
αSMA
EEF2

## Slide 2
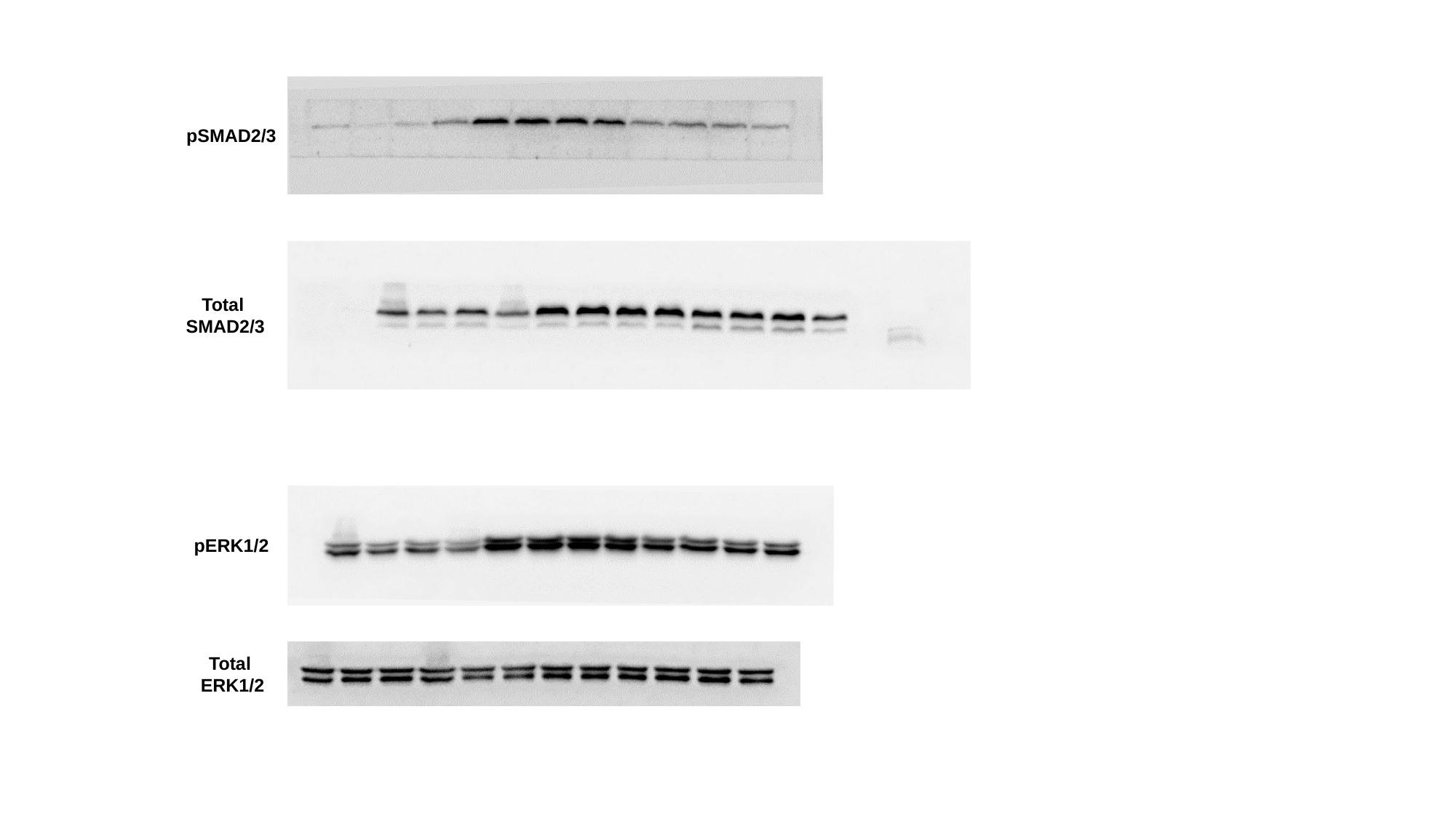

pSMAD2/3
Total
SMAD2/3
pERK1/2
Total
ERK1/2

Supplement: Supplementary file 1 — (PPTX 317 kb) [file 210_2020_1998_MOESM1_ESM.pptx]
